# Crystal structures of MdfA complexed with acetylcholine and inhibitor reserpine

**DOI:** 10.1007/s41048-016-0028-1

**Published:** 2016-10-12

**Authors:** Ming Liu, Jie Heng, Yuan Gao, Xianping Wang

**Affiliations:** 1 College of Biotechnology, Tianjin University of Science and Technology, Tianjin, 300457 China; 2 National Laboratory of Macromolecules, National Center of Protein Science - Beijing, Institute of Biophysics, Chinese Academy of Sciences, Beijing, 100101 China

**Keywords:** MdfA, Reserpine, DHA12, Antiporter, Acetylcholine

## Abstract

The DHA12 family of transporters contains a number of prokaryotic and eukaryote membrane proteins. Some of these proteins share conserved sites intrinsic to substrate recognition, structural stabilization and conformational changes. For this study, we chose the MdfA transporter as a model DHA12 protein to study some general characteristics of the vesicular neurotransmitter transporters (VNTs), which all belong to the DHA12 family. Two crystal structures were produced for *E. coli* MdfA, one in complex with acetylcholine and the other with potential reserpine, which are substrate and inhibitor of VNTs, respectively. These structures show that the binding sites of these two molecules are different. The Ach-binding MfdA is mainly dependent on D34, while reserpine-binding site is more hydrophobic. Based on sequence alignment and homology modelling, we were able to provide mechanistic insights into the association between the inhibition and the conformational changes of these transporters.

**Electronic supplementary material:**

The online version of this article (doi:10.1007/s41048-016-0028-1) contains supplementary material, which is available to authorized users.

## Introduction

MdfA, as a typical antiporter of the major facilitator superfamily (MFS), has been the subject of extensive study, especially in the research of multidrug transport mechanisms (Edgar and Bibi 1997). Many small molecules are substrates of MdfA, including neutral compounds such as chloramphenicol (Cm) and thiamphenicol, lipophilic cations such as tetraphenylphosphonium (TPP^+^) and ethidium bromide (EtBr), and the zwitterionic drug—ciprofloxacin (Adler and Bibi 2004). Crystal structures of *E. coli* MdfA (ecMdfA) have recently been reported by our laboratory (Heng et al. 2015). Based on the parsed structures of ecMdfA, the key location for the binding of a variety of substrates was determined to be the large central cavity within MdfA, which is lined with mostly hydrophobic residues. An acidic residue, D34, is also present deep within this cavity, and is proposed to be critical for the binding of certain substrates (Heng et al. 2015). Also, the substrate-bound crystal structure of MdfA provided a base for further structural and functional study of other homologous proteins, such as MdtM, another MFS transporter, which share high sequence identity with MdfA (Paul et al 2014).

In mammalian cells, vesicular monoamine transporter (VMAT) and vesicular acetylcholine transporter (VAChT) are homologous proteins with MdfA and transport mono-positively charged amine neurotransmitters and hormones from the cytoplasm and concentrate them within secretory vesicles by alternating their access mechanism (Parsons 2000). They are sub-classified into the SLC18 group of MFS in eukaryotes (Lawal and Krantz 2013) and also belong to the DHA12 family (Paulsen et al. 1996; Putman et al. 2000; Heng et al. 2015). Two isoforms of VMAT, 1 and 2, have been cloned and identified (Erickson et al. 1992). VMATs and VAChT are predicted to possess 12 transmembrane helices (TM) consisting of two pseudo-symmetrical domains, and use the proton motive force (PMF) of the transmembrane electrochemical proton gradient as the driving force for the uptake of cytoplasmic substrates into the vesicles (Zhang et al. 2015). Extensive pharmacological studies have shown that VMATs and VAChT have broad substrate specificity, and a large number of substrates and inhibitors of VMATs and VAChT have been identified (Yelin and Schuldiner 1995; Erickson et al. 1996; Bravo et al. 2005). Several residues in these proteins have been identified to contribute to substrate recognition (Merickel et al. 1995; Finn and Edwards 1997), protonation and energy coupling (Yaffe et al. 2013).

To study the transport mechanisms of VMATs and VAChT, a homology model based on LacY was initially used to investigate the alternating access mechanism of VMAT2 (Vardy et al. 2004; Yaffe et al. 2013). However, owing to their low sequence similarity and the fact that LacY is a symporter rather than antiporter, LacY is not suitable for studying the proton/substrate antiport mechanism of VMATs and VAChT. Unlike previous passable symporter LacY, it is reasonable to assume that the proton/substrate antiporter MdfA would be a better reference for homology model and functional study of VMATs and VAChT. Although MdfA and its eucaryotic homologs share low sequence identity (Fig. S1), those conserved motifs sharing in DHA12 family reflect the fact that they possess similar proton-substrate antiporting mechanism as discussed at length in previous paper(Heng et al. 2015). More importantly, several well-known substrates of MdfA are also transported by VAChT, including tetraphenylphosphonium, ethidium and rhodamine (Bravo et al. 2005). An inhibitor of VMATs, reserpine (Erickson et al. 1996), is also reported to inhibit the translocation cycle of MdfA (Edgar and Bibi 1999).

Reserpine is an antipsychotic and antihypertensive drug used for the relief of psychotic symptoms, and irreversibly blocks VMATs in the presynaptic neurons (Preskorn 2007). Reserpine was proposed to directly bind and competitively inhibit the efflux pump during proton/substrate antiport (Holler et al. 2012).

**Fig. 1:**
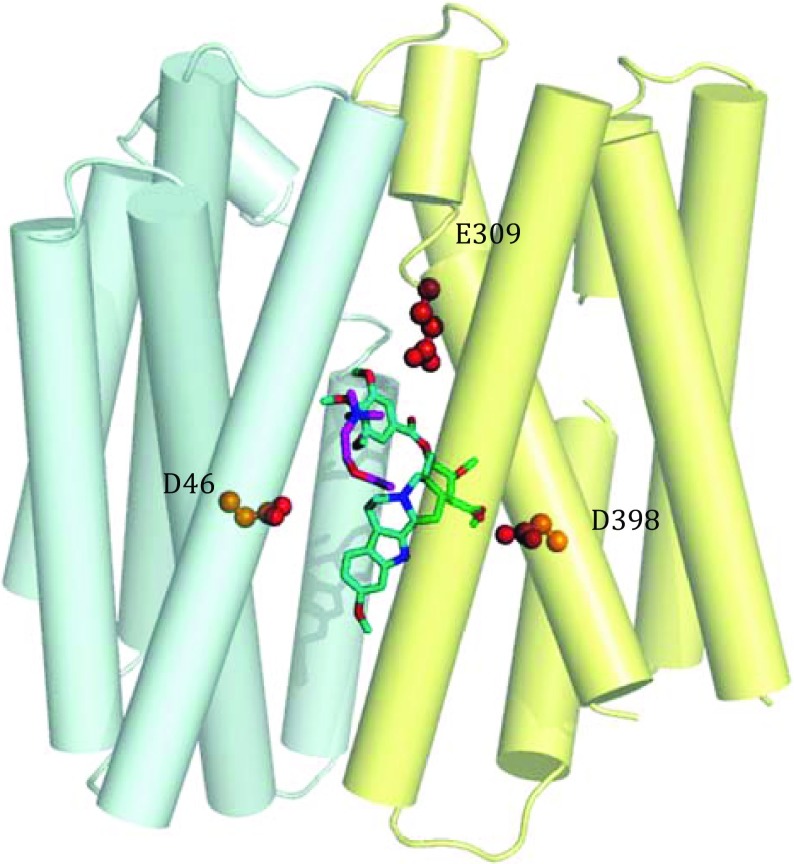
Possible inhibition mechanism for VAChT. Three conserved, negatively charged residues of human VAChT (D46, E309 and D398) line the pocket of the predicted VAChT structure. ACh and reserpine are, respectively, represented with *magenta* and *cyan* sticks. The N-terminal domain is shown in *blue*, and the C-terminal domain is shown in *yellow*

To elucidate the transportation mechanism of VMATs and VAChT and the potential inhibition mechanism of reserpine towards multiple transporters, we therefore used the ecMdfA structure as a model to investigate the binding of acetylcholine (ACh) and reserpine molecules. Here, we report the crystal structures of ecMdfA complexed with ACh at 2.8-Å resolution using the soaking method, and with possible reserpine at 3.5-Å resolution using co-crystallization. Both of these structures were captured in the inward-facing conformation, albeit under different crystallization conditions. The reserpine-MdfA complex structure shows more extensive interactions between the transporter and substrate than that observed within the ACh-MdfA complex. Based on these ecMdfA structures, we could simulate the docking of reserpine into a model of VAChT (Fig. 1). We used this docked model to postulate a mechanism for substrate transport and inhibitor binding for VAChT.

## Results

### Acetylcholine (ACh)-bound structure of ecMdfA

**Fig. 2:**
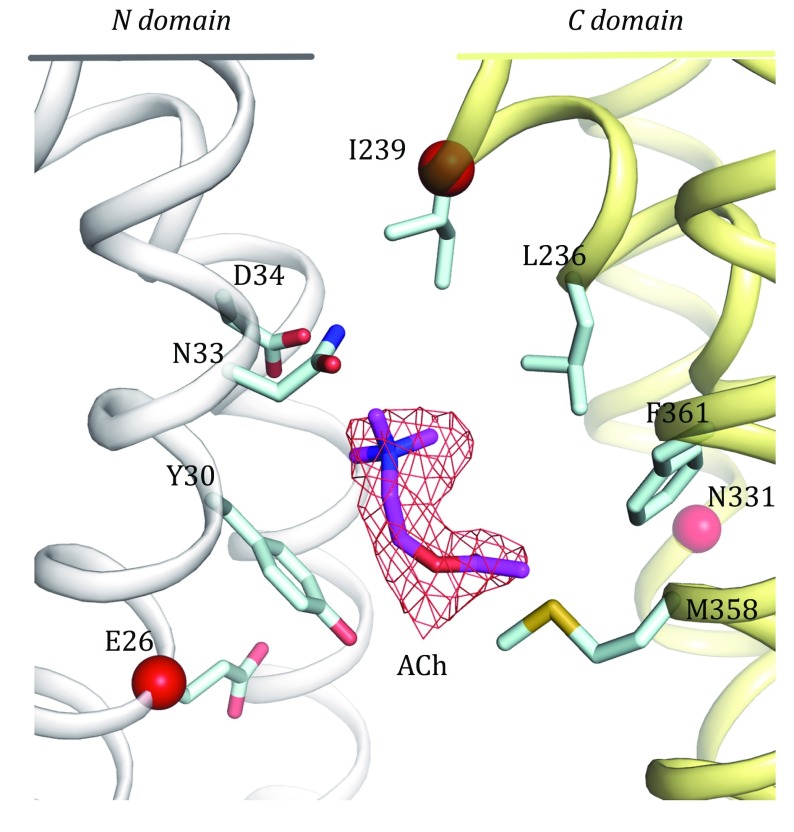
ACh-binding sites of MdfA. ACh (*magenta sticks*) binds in the pocket between the N- and C-terminal domains, which are illustrated in *white* and *yellow*, respectively. The omit Fo-Fc density (in *red*) for ACh is contoured at 3*σ*. Three equivalent conserved acid residues which may be important in substrate binding and protonation in VAChT are marked with *red spheres*

The complexed structures of ecMdfA with ACh and reserpine were obtained under two distinct crystallization conditions. Their space groups were *C2* and *P3*_*1*_ *21,* respectively, and the structures were resolved with the molecular replacement method (Table S1). In the two crystal structures, the final refined structural models contained the intact peptide chains of residues 10–400 and 14–400, respectively, and were both in the inward-facing conformation consistent with the previously reported Dxc-ecMdfA structure (Heng et al. 2015). The ACh-complex structure was obtained with the soaking method, during which the pH value was increased from 5.4 to 8.0, using the same conditions under which crystals of Dxc-ecMdfA were obtained and supplemented with 5 mmol/L ACh. We postulated that Dxc could be replaced by ACh when the pH of crystallized condition increases since key amino acids around the binding cavity determine ecMdfA prefers to bind positively charged Ach rather than negatively charged Dxc. Results of the structural studies showed no differences between these two complexed structures, except for the electron density of Dxc being replaced with that of ACh (Fig. 2).

As expected, the resolved ACh-complex structure shows that ACh indeed binds in the vicinity of the D34 residue. The distance between the positively charged head group of ACh and the negatively charged side chain of D34 is ~4.5 Å, while residues Y30, L263, F358 and M358 form hydrophobic interactions with ACh (Fig. 2). We overlaid the potential protonation sites of the VMATs or VAChT on the structure of ecMdfA based on sequence alignment (Fig. S1). We found that three acidic residues of VAChT, D33, E309 and D398, correspond with residues E26, I239 and N331 of ecMdfA, respectively, which are located near the ACh-binding pocket. E309 is located at the bottom of the substrate-binding cavity, and may play the same critical protonation-deprotonation role as D34 in ecMdfA. Residue D33, which corresponds with E26 on ecMdfA, has been shown to take part in the recognition of positively charged substrates (Merickel et al. 1995).

### Reserpine-bound structure of ecMdfA

The second crystal structure we generated was of reserpine-complexed ecMdfA. Reserpine is an effective inhibitor of MdfA (Edgar and Bibi 1999). There were two ecMdfA molecules in one asymmetric unit of the *P3*_*1*_ *21* structure. The interfaces are consisted of the twelfth helices of ecMdfA, and comprised mostly hydrophobic amino acids, such as L382, V386 and I389. Both of the two transporters in the unit adopted an inward-facing conformation (Fig. S2).

**Fig. 3:**
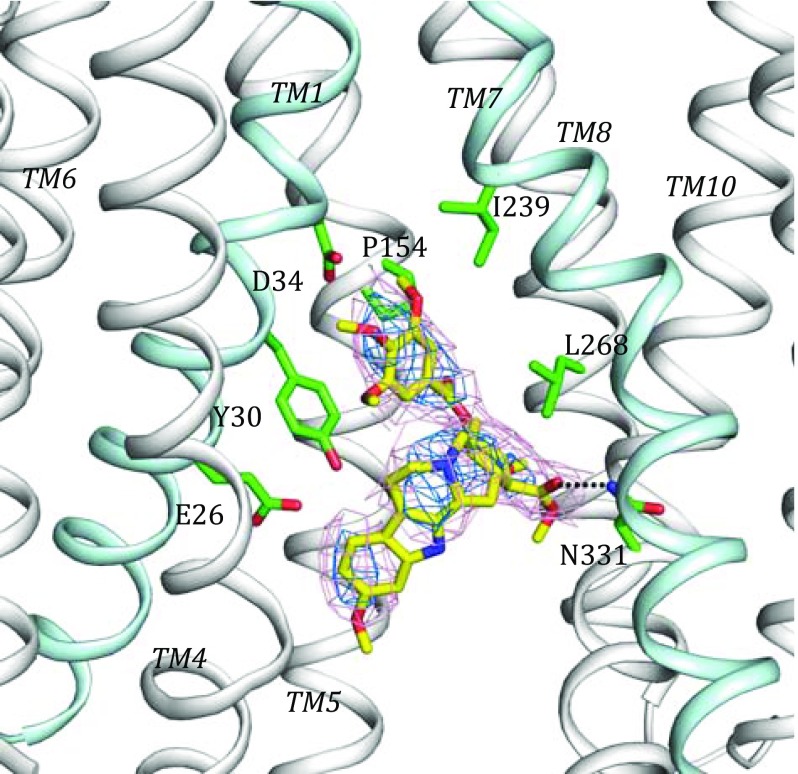
Reserpine-binding site of MdfA. The backbone of MdfA is shown in cartoon representation. Two important cavity helices, TM1 and TM7, are coloured in *cyan*, while the other helices are in *white*. The reserpine molecule is illustrated with *yellow sticks*. Amino acid residues near reserpine are shown with *green sticks*. The omit 2Fo-Fc density for reserpine is contoured in *marine* (1*σ*) and *pink* (0.4*σ*). Hydrogen bonds between reserpine and N331 are shown as *dotted lines*

**Fig. 4:**
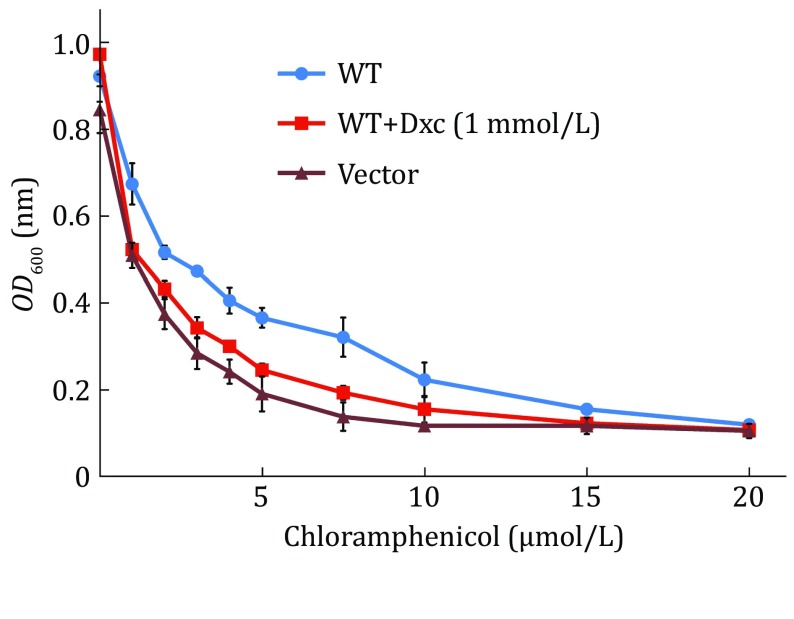
Inhibition of MdfA chloramphenicol resistance by Dxc. *E. coli* C43 (DE3) cells harbouring wild-type MdfA or vector only were grown in the presence of increasing concentrations of chloramphenicol supplemented with Dxc (1 mmol/L). Relative growth was calculated from the cell density and measured by culture absorption at 600 nm. Assays were done in quadruplicate, plotting the average with error bars of ±1 SD

In this structure, we identified a suspected density attributable to the presence of reserpine in the central cavity. We built a reserpine molecule into the model at this position to analyse its possible inhibition mechanism. Reserpine occupies a more hydrophobic position than ACh because it is a larger molecule. The trimethoxybenzoyl in the tail of reserpine is near residue D34. Hydrophobic interactions were localized on the trimethoxybenzoyl group and other groups of the reserpine molecule (Fig. 3). For comparison, in the previous 2.0-Å resolution Dxc-complex structure of ecMdfA, residue D34 was observed to form hydrogen bond with Dxc, which exerts an inhibitory effect on the Cm resistance of ecMdfA (Fig. 4). In contrast, three methoxyls of reserpine were in proximity to the D34 residue, potentially forming hydrogen bonds for structure stabilization. In addition, both Dxc and reserpine directly interact with residue P154 in the conserved motif C of MdfA. The mechanism of reserpine inhibition is therefore likely to be associated with motif C.

## Discussion

According to the multiple sequence alignment of the 12 transmembrane proton-dependent bacterial multidrug transporters of the MFS family (also known as DHA12), these transporters contain a number of highly conserved motifs, which indicates that they may share a similar transportation mechanism (Putman et al. 2000). Here, we show the sequence alignments of VMATs and VAChT from *Homo sapiens*, *Mus musculus* and *Rattus norvegicus* (Fig. S1), including their conserved motifs. Motif A is in the cytoplasmic loop between TM 2 and 3 of the MFS, with the most conserved residue being G73, which has previously been clearly demonstrated in the structure of YajR (Jiang et al. 2013). Motif C, motif D and motif G (or motif C′) all contain a conserved proline residue in their consensus sequences of TMs 5, 1 and 11, respectively. Proline, which is incapable of acting as a hydrogen bond donor, often plays a role of helix “breaker” in the transmembrane helices of α-helical membrane proteins (Chandrasekaran et al. 2006).

Based on the structures of the ecMdfA-ACh and ecMdfA-reserpine complexes, we modelled a homology structure of VAChT overlaid with ACh and reserpine (Fig. 1). The homology VAChT model was cytoplasm-facing and consisted of 12 transmembrane helices, similar to the structure of ecMdfA. In addition, there were three carboxyl residues, D46, E309 and D398, located in TM1, 7 and 8, respectively, all within the central cavity. In the ACh-VAChT complexed model, ACh binds to E309 through negative–positive charge interaction. Residue E309 is located at the bottom of the large hydrophobic substrate-binding pocket, corresponding with the positioning of residue D34 of ecMdfA. This confirmed that D34 of MdfA is likely deprotonated during the process of substrate binding as previously proposed (Heng et al. 2015), as upon ligand binding, E309 is likely to become similarly deprotonated. This buried acidic residue is necessary for the recognition of monovalent, positively charged substrates. In the VAChT-reserpine-complexed model, it appeared that residues D398 and E309 may form a hydrogen bond network with reserpine. This observation may explain the inhibition mechanism of reserpine, because this hydrogen bond network would stabilize VAChT in the cytoplasm-facing conformation, whereby inactivating it (Darchen et al. 1989). Furthermore, Khare et al. (2010) showed that both D398 and E309 directly bind with the allosteric inhibitor—vesamicol, but only the E309 residue can bind ACh, while D398 does not. These observations are consistent with our model.

**Fig. 5:**
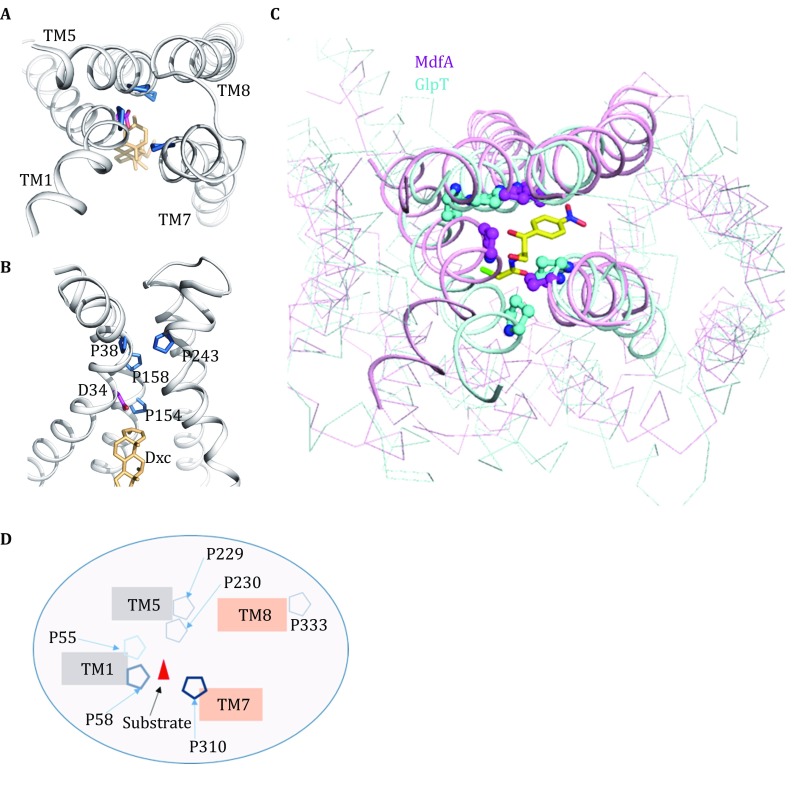
Structural features of the 3D-motif C of MdfA and a schematic of the transportation model. **A** 3D-motif C of MdfA (PDB ID: 4ZP0) viewed from the periplasmic side. Prolines are shown as *blue sticks* and Dxc is in *yellow*. **B** 3D-motif C of MdfA viewed parallel to the membrane. **C** Superposition of the 3D-motif C of MdfA and GlpT. **D** Schematic of the 3D-motif C of mVAChT

Most members of the DHA12 family recognize multiple substrates and trigger conformational change upon substrate binding. Why the binding of a variety of substrates can drive the transportation cycle, while inhibitor binding may stabilize them in only one of their two possible conformations. The conserved proline in VMATs and VAChTs offers a clue as to the mechanism of substrate binding and the subsequent conformational change exerted. Highly conserved prolines are located in motif C, which is also known as the “antiporter motif” (De Jesus et al. 2005). Several conformationally sensitive residues, including some prolines, have been identified in motif C in VMAT2 (Ugolev et al. 2013) and VAChT (Luo and Parsons 2010). Here, we presented structural information of motif C from ecMdfA (Fig. 5A, B). Several prolines from TM1, 5 and 7 were observed to cluster together to form a “bottleneck” in the 3D structure. Similar features of other DHA12 members have been shown to inhibit solute leak inside the cell (De Jesus et al. 2005). We named this structural motif “3D-motif C”, which consists of four helices each from the N-terminal and C-terminal domains. We also found another inward-facing MFS antiporter GlpT, an organic phosphate/inorganic phosphate antiporter, which contained a similar prolines cluster in the same location of antiporter motif when GlpT was superposed on the ecMdfA structure (Huang et al. 2003) (Fig. 5C). Molecular dynamics simulations of GlpT are consistent with the proposed mechanism that proline-induced flexibility in the TM helices is critical to the conformational change of MFS pseudo-rigid body motions (D’Rozario and Sansom 2008). Based on this, a sketch map of the 3D-motif C in mVAChT (mouse VAChT) could be postulated on the basis of multiple sequence alignment and homology modelling (Fig. 5D). Residue P333 of mVAChT differs from 3D-motif C in MdfA and may facilitate the bending of TM 8 towards the proline cluster, similarly to the role of P263 in MdfA. Mutations near 3D-motif C of VMATs have been shown to influence the transport rate by affecting the rate-limiting step of the transport cycle (Ugolev et al. 2013). Evidence of interactions between P154 and inhibitors in the ecMdfA-Dxc and ecMdfA-reserpine complexes points to the close association of 3D-motif C with inhibition. In short, the conserved sequence of 3D-motif C may participate in the gating-like movements of these transporters.

In conclusion, the structures reported here of ecMdfA in complex with ACh and potential reserpine improve our understanding of the mechanisms of multiple substrate recognition of MdfA. Using sequence alignment of the VMATs, VAChT and ecMdfA, several conserved residues and motifs were identified. Therefore, we were able to model a homology structure of VAChT based on the ecMdfA structure, and the model had credibility based on previously published biochemical results. The 3D-motif C was determined to likely play a critical role in substrate transport and conformational change. Study of ecMdfA-complexed structures and predicted models may facilitate further research on the structure and function of vesicular neurotransmitter transporters. Certainly, the authentic structures of VNTs are necessary to explain the mechanisms of substrates recognition and transport.

## Materials and methods

### Protein expression and purification

The full-length *MdfA* gene was subcloned into the pET-28a vector (Novagen) with a C-terminal His_6_-tag(Heng et al. 2015). For protein expression, *E. coli* C43(DE3) strain transformed with the recombinant plasmid was cultured in Terrific Broth supplemented with 25 μg/mL kanamycin at 37 °C and induced at an *OD*_600nm_ of 0.8 with 0.5 mmol/L isopropyl-d-thiogalactoside (IPTG) at 16 °C for 18 h. The cells were harvested by centrifugation at 4000 *g* for 30 min, resuspended in buffer A (20 mmol/L HEPES pH 7.2, 300 mmol/L NaCl, 10% (*v*/*v*) glycerol and 5 mmol/L β-mercaptoethanol) and then disrupted at 10,000–15,000 psi using a JN-R2C homogenizer (JNBio, China). Cell debris was removed by centrifugation at 17,000 *g* for 15 min. The supernatant was ultracentrifuged at 100,000 *g* for 1 h. Membrane fraction was harvested and solubilized with 0.5% (*w*/*v*) *n*-decyl-β-d-maltopyranoside (DM; Anatrace) for 15 min at 4 °C. We eluted the target proteins from 2 mL Ni^2+^-nitrilotriacetate affinity resin (Ni–NTA; Qiagen) using 15 mL buffer A containing 300 mmol/L imidazole and 0.2% (*w*/*v*) DM, and concentrated to about 10–15 mg/mL. The concentrated sample was incubated with 0.8 mmol/L Cm and subsequently loaded onto a Superdex-200 10/30 column (GE Healthcare) pre-equilibrated with buffer B (20 mmol/L HEPES pH 7.2, 100 mmol/L NaCl and 5 mmol/L β-mercaptoethanol), supplemented with mixed detergents of 0.2% (*w*/*v*) *n*-nonyl-β-d-glucopyranoside (NG; Anatrace) and 0.025% (*w*/*v*) *n*-dodecyl-*N*,*N*-dimethylamine-*N*-oxide (LDAO; Anatrace). Cm (0.8 mmol/L) was added to the collected protein, which was then concentrated to 20 mg/mL and mixed with 0.5 mmol/L reserpine for crystallization.

### Crystallization

Crystal screening was performed using the hanging drop vapour-diffusion method (1 µL plus 1 µL over 200 µL) at 16 °C and obtained under the conditions of 0.1 mol/L Tris (pH 8.0), 0.22 mol/L sodium citrate and 35% (*v*/*v*) PEG400 from the MemGold screening kit (molecular dimensions). The crystals grew in ~2 months and were flash-cooled in liquid nitrogen for storage and data collection.

### Data collection and structure determination

X-ray diffraction datasets were collected at Shanghai Synchrotron Radiation Facility (SSRF) and processed with the HKL-2000 software package (Otwinowski and Minor 1997). The space group of the reserpine structure was *P3*_*1*_ *21*, while the space group of ACh was *C2*. All ligand-complex structures were resolved by molecular replacement. There were two MdfA molecules per crystallographic asymmetric unit for *P3*_*1*_ *21*. The model was further refined using the program Coot (Emsley and Cowtan 2004). Model validation was carried out using the web-based program MolProbity (Davis et al. 2004).

### Inhibition of drug resistance assays

The *E. coli* C43 (DE3) strain was transformed with the *MdfA* gene-containing pET28a plasmid. A single clone was picked from LB-agar plates for inoculation into 5 mL LB supplemented with kanamycin (30 μg/mL) and grown at 37 °C. The cultures were induced with 0.5 mmol/L IPTG once an *OD*_600nm_ of 0.6 was obtained, and the cultures were incubated overnight for protein expression. The cells were then diluted into 48-well plates containing 1 mL LB with increasing concentrations of the test drug (Cm) and kanamycin (30 μg/mL). At the beginning of each typical experiment, the cell density in the wells was 0.05 *OD*_600nm_ units. Plates were incubated at 37 °C with shaking, and the cell density was measured with a Varioskan Flash reader (Thermo Fisher Scientific) by following the absorption at 600 nm over 12 h.

## Electronic supplementary material

Below is the link to the electronic supplementary material.

Supplementary material 1 (DOC 602 kb)

## Abbreviations

Ach: Acetylcholine
Cm: Chloramphenicol
Dxc: Deoxycholate
TM: Transmembrane (helix)
