# Supplementary material for: Crystal structures of MdfA complexed with acetylcholine and inhibitor reserpine
Source: Biophys Rep. 2016 Oct 12;2(2):78–85. doi: 10.1007/s41048-016-0028-1 (PMC5138259; doi:10.1007/s41048-016-0028-1)
Supplement: Supplementary file 1 — Supplementary material 1 (DOC 602 kb) [file 41048_2016_28_MOESM1_ESM.doc]

# Supplementary materials


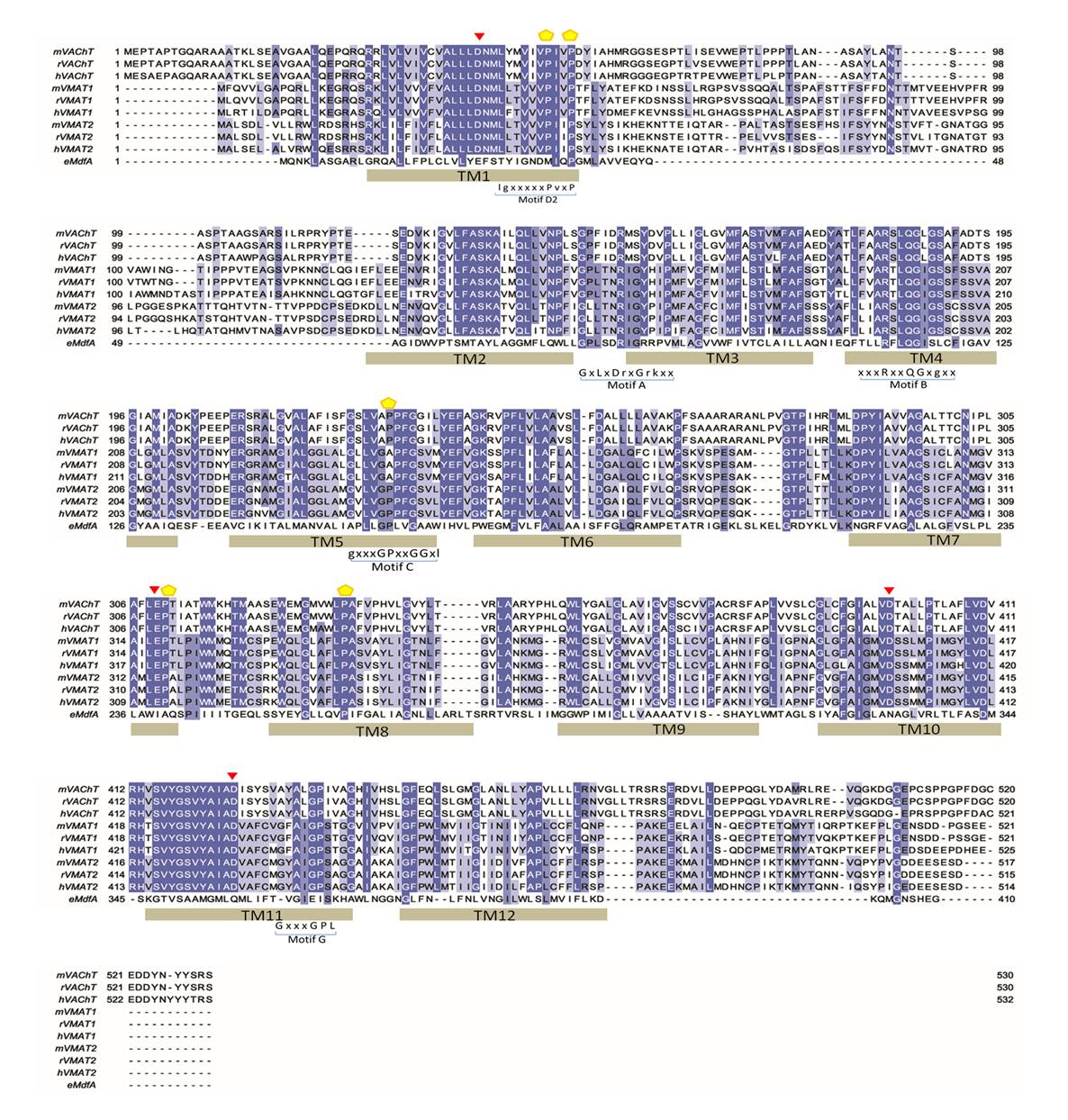


## Supplementary Figure S1. Sequence alignment of MdfA, VMAT and VAChT

Sequence alignment of VMAT1, VMAT2 and VAChT from *Homo sapiens*, *Mus musculus*, *Rattus norvegicus*, and the MdfA protein from *E. coli.* Consensus sequences of the listed conserved motifs were adapted from the review by Putman *et al*. (Putman et al, 2000). Red triangles indicate negatively charged residues in the binding cavity located in the middle of the TMs. The yellow sticks indicate the several conserved proline residues which may participate in forming the “3D-antiporter motif”. Sequences were aligned with the program ClustalX (Larkin et al, 2007) and formatted with JalView (Waterhouse et al, 2009).

## Supplementary Table S1. Statistics of the diffraction and structure refinement data

| **Crystal** |  | | **MdfA-reserpine** | **MdfA-ACh** |
| --- | --- | --- | --- | --- |
| Data processing | |  | | |
| Beamline | | | BL17U at SSRF | BL17U at SSRF |
| Wavelength (Å) | | | 1.0000 | 1.0000 |
| Space group | | | P3_1_21 | C2 |
| Cell dimensions | | |  |  |
| a, b, c (Å) | | | 86.3, 86.3, 326.1 | 95.0, 65.1, 108.4 |
| α, β, γ (º) | | | 90, 90, 120 | 90, 110.6, 90 |
| Resolution (Å)^a^ | | | 50‒3.5 (3.5–3.6) | 50‒2.8 (2.9–2.8) |
| Completeness (%) | | | 97.9 | 95.3 (96.0) |
| R_merge_ (%) | | | 12.0 (>100) | 12.3 (92.3) |
| CC_1/2_ | | | 0.995 (0.715) | 0.997 (0.760) |
| I /σ(I)  Wilson *B-*factor | | | 15.0 (2.0)  144 | 12.8 (1.7)  74 |
| Unique reflections | | | 17,980 (1,767) | 14,606 (1,470) |
| Redundancy | | | 4.6 (4.5) | 4.6 (4.4) |
| Refinement | |  | | |
| Resolution (Å) | | | 50–3.5 | 50–2.8 |
| Number of reflections (test)^b^ | | | 17,954 (1,767) | 14,589 (699) |
| R_work_ / R_free_ (%) | | | 29.2/32.9 | 22.4/25.5 |
| Average *B-*factor (Å^2^) | | |  |  |
| Protein | | | 136 | 62 |
| Ligand (No. of atoms) | | | 164 (44) | 78 (10) |
| Water (No. of atoms) | | | 117 (1) | 51 (4) |
| R.m.s.d. from ideal geometry | | |  |  |
| Bond lengths (Å) | | | 0.004 | 0.002 |
| Bond angles (º) | | | 1.007 | 0.753 |
| Ramachandran plot (%)^c^ | | |  |  |
| Favored region | | | 95.1 | 97.7 |
| Allowed region | | | 4.6 | 2.3 |

^a^. Values in parentheses are for shells with the highest resolution.

^b^. All structures were refined with the same “test” set of R_free_ reference reflections.

^c^. Calculated using MolProbity.


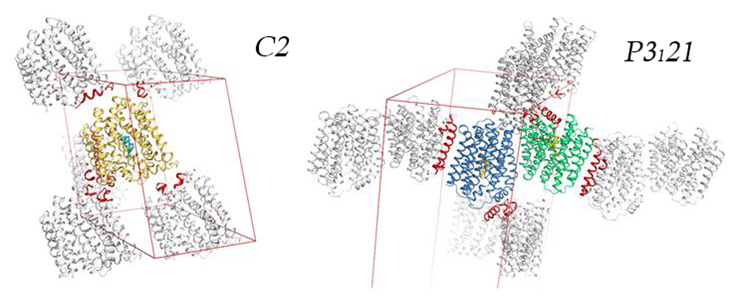


## Supplementary Figure S2. Crystal packing in the two different space groups

The crystal packing details in the *C2* and *P3_1_21* space groups. a. One MdfA molecule is contained in an asymmetrical unit in *C2*. Every molecule interacts with five MdfA via several loop regions. b. Two MdfA molecules are contained in an asymmetrical unit in *P3_1_21*. The crystal packing was mainly mediated by TM6 and the amphipathic helices (α6 and α7).

# References

**Putman M, van Veen HW & Konings WN (2000) Molecular properties of bacterial multidrug transporters. *Microbiol Mol Biol Rev* 64: 672-693**

**Larkin MA, Blackshields G, Brown NP, Chenna R, McGettigan PA, McWilliam H, Valentin F, Wallace IM, Wilm A, Lopez R, Thompson JD, Gibson TJ & Higgins DG (2007) Clustal W and Clustal X version 2.0. *Bioinformatics* 23: 2947-2948**

**Waterhouse AM, Procter JB, Martin DM, Clamp M & Barton GJ (2009) Jalview Version 2--a multiple sequence alignment editor and analysis workbench. *Bioinformatics* 25: 1189-1191**
